# Infection hospitalisation in systemic lupus in Sweden

**DOI:** 10.1136/lupus-2021-000510

**Published:** 2021-09-14

**Authors:** Julia F Simard, Marios Rossides, Iva Gunnarsson, Elisabet Svenungsson, Elizabeth V Arkema

**Affiliations:** 1 Department of Epidemiology & Population Health, Stanford University School of Medicine, Stanford, California, USA; 2 Clinical Epidemiology Division, Department of Medicine Solna, Karolinska Institutet, Stockholm, Sweden; 3 Department of Medicine Solna, Rheumatology Unit, Karolinska Institutet, Stockholm, Sweden

**Keywords:** lupus erythematosus, systemic, epidemiology, therapeutics, antirheumatic agents

## Abstract

**Objective:**

Immune dysregulation in SLE and the corresponding immune-modulating and immunosuppressive nature of the treatments may play key roles in infection risk. We compared serious infection rates among individuals with incident SLE with the general population, and examined the role of treatment initiation in SLE.

**Methods:**

Newly diagnosed patients with SLE (2006–2013) and general population comparators from the Swedish Lupus Linkage cohort were followed for serious infection through 2016. Adjusted Cox and frailty models estimated the relative risk of first and recurrent infections, respectively. Using a new-user design, rates of serious infections were compared between disease-modifying antirheumatic drugs (DMARDs) and hydroxychloroquine (HCQ) initiators. We then evaluated three DMARDs (azathioprine, mycophenolate mofetil and methotrexate) in multivariable-adjusted models.

**Results:**

Individuals with SLE experienced more infections (22% vs 6%), especially during the first year of follow-up, and recurrent serious infections were also more common (HR=2.22, 95% CI 1.93 to 2.56). DMARDs were associated with a higher rate of serious infection versus HCQ (HR=1.82, 95% CI 1.27 to 2.60), which attenuated after multivariable-adjustment (HR=1.30, 95% CI 0.86 to 1.95). Among DMARDs, azathioprine was associated with infection (HR=2.19, 95% CI 1.14 to 4.21) and mycophenolate mofetil yielded an HR=1.39 (95% CI 0.65 to 2.96) in multivariable-adjusted models compared with methotrexate. Results were comparable across numerous sensitivity analyses.

**Conclusion:**

Individuals with incident SLE were 2–4 times more likely to be hospitalised for infection and experienced more recurrent infections than the general population. Among DMARD initiators, azathioprine was associated with the highest rate.

**Key messages**

**What is already known about this subject?**

- The immune-modulating and immunosuppressive aspects of SLE and its treatment have been implicated in higher risk of infection in cohorts of patients with SLE, although most published findings to date have focused on prevalent SLE.

**What does this study add?**

- Individuals recently diagnosed with SLE were more likely to be hospitalised with an infection and to have recurrent serious infections compared with age-matched and sex-matched general population comparators.
- The rate of infection was pronounced in the year following diagnosis and remained elevated for the study period compared with the general population.
- Among individuals with SLE initiating azathioprine, mycophenolate or methotrexate, azathioprine initiators consistently experiencing more serious infections compared with the methotrexate initiators.

**How might this impact on clinical practice or future developments?**

- Given availability of immunisations for pneumonia and a considerable proportion of pneumonia infections in these patients with lupus, future work needs to examine vaccine uptake and effectiveness in this population, as well as examine antibody response following vaccination.
- Initiation of azathioprine was consistently associated with increased rates of serious infection in this inception cohort compared with methotrexate initiators, which may warrant additional counselling about infection-related risks in these patients.

## Introduction

Dysregulation of the immune system in SLE, and the corresponding immune-modulating and immunosuppressive nature of the treatments, may play key roles in infection risk. Whether due to decreased phagocytosis, impaired B and T cell function or complement deficiency, numerous studies found that patients with SLE are vulnerable to infection.^1 2^

Serious infections contribute to hospitalisation and death in patients with SLE.^3–8^ Treatments such as cyclophosphamide and corticosteroids have been linked to serious infections in patients with lupus, however less is known about disease-modifying therapies such as azathioprine and mycophenolate mofetil (MMF).^9–11^ Even a recent systematic review found considerable heterogeneity by medication and across studies.^12^ Despite the complex immunosuppressive treatment strategies and dynamic nature of SLE, little is known about the risk of infection as a function of time since diagnosis or with respect to medication.

This work evaluated risk periods of serious infection, defined as primary cause of hospitalisation, focusing on timing of infection after diagnosis and treatment and comparing with a matched general population comparator for baseline age-specific and sex-specific risks. We estimated the rate of serious infection in the SLE population overall and by time since diagnosis, and then estimated the relative risk of serious infection comparing initiation of different treatment strategies in SLE.

## Methods

### Study population

This cohort study leveraged the Swedish Lupus Linkage (SLINK) of prospectively collected data from the Swedish National and Quality Registers.^13^ The linkage includes data from numerous registers including healthcare utilisation, pharmacy dispensings, inpatient admissions, births, cancer, mortality and causes of death. By virtue of the national public healthcare system, all residents and citizens with a personal identification number are eligible to receive care and can be linked across the tax-funded system.^14^

Incident SLE was defined via the National Patient Register (NPR), which includes all inpatient admissions from 1987 onwards and nearly all non-primary outpatient specialist care since 2001. Individuals aged 18–85 years were considered to have SLE if they had ≥2 visits listing an SLE International Classification of Diseases (ICD) code in the NPR with ≥1 diagnosis by a specialist who typically manages or diagnoses SLE. The date of inclusion into the cohort was the date these criteria for SLE were met (index date). To restrict to an incident cohort, we required the first diagnosis of SLE to occur in 2006 or later, which provided ≥5 years of NPR data to identify and exclude prevalent SLE.

A non-SLE comparison group from the general population was identified from the Total Population Register. Five comparators were matched to each individual with incident SLE on birth year, sex and region of residence and were required to be living in Sweden at the date of the matched case’s date of inclusion (index date).

### Medication ascertainment

Medication exposures were primarily identified from the Prescribed Drug Register (PDR), which captures all prescription dispensings from Swedish pharmacies since mid-2005. Using a new-user design, we identified those in the incident SLE group without any history of hydroxychloroquine (HCQ) who initiated HCQ during follow-up. We also identified DMARD initiators (methotrexate, MMF and azathioprine) without any prior DMARD, with or without a history of HCQ. Eighty-seven per cent of DMARD initiators had multiple dispensings of their initial DMARD. Very few patients were dispensed tacrolimus, ciclosporin, cyclophosphamide, sulfasalazine or leflunomide as their first DMARD (n=9), and due to sparse data, these groups were excluded from treatment analyses. Medications given in the hospital via infusion are not included in the PDR. Because of the new-user active comparator design, the majority of patients were excluded because they had a history of DMARDs (n=566) or HCQ (n=826) before start of follow-up. Over 500 had no DMARD dispensation, 5 had a biologic DMARD (that could be identified in our data) and a small number started DMARD after the end of study.

### Outcomes

The primary outcome of interest was serious infection, which was defined as hospitalisation with infection as the primary diagnosis using ICD codes from the NPR inpatient component.^3 8 15^ When possible, we subdivided into pneumonia, sepsis and other opportunistic infections, which included: herpes zoster, pneumocystosis, progressive multifocal leukoencephalopathy, legionellosis, coccidioidomycosis, histoplasmosis, non-tuberculosis mycobacteria, salmonellosis, nocardiosis, blastomycosis, cryptococcosis, aspergillosis, listeriosis and toxoplasmosis.

### Follow-up

For the analysis of infection rates in SLE compared with the general population, start of follow-up was the index date and end of follow-up was first of: date of infection hospitalisation, first emigration, death or 31 December 2016. A recurrent infection was defined as present if a patient had a hospital admission date at least 31 days after the previous admission to be considered a new (independent) infection. For recurrent infection outcomes, individuals exited the study at death, first emigration or the end of the study.

For the analysis of infection rates comparing treatments, individuals contributed person-time in the HCQ-initiator group from first HCQ dispensation until first dispensation of any DMARD, start of a biologic, death, infection, emigration or 31 December 2016. Individuals contributed person-time in the DMARD-initiator group from first ever DMARD dispensing until biologic initiation, dispensation of the excluded DMARDs (ciclosporin, cyclophosphamide, sulfasalazine or leflunomide), death, infection, emigration or 31 December 2016. Biologics use was identified in the Swedish Rheumatology Register and the PDR (see online supplemental table 1).

10.1136/lupus-2021-000510.supp1 Supplementary data

### Additional covariates

Age, date of emigration and country of birth (Sweden vs non-Sweden) were obtained from the Total Population Register. Highest level of completed education was collected from the Longitudinal Integrated Database for Health Insurance and Labor Market Studies. Comorbid conditions, including diabetes, nephritis and history of infection were abstracted using ICD codes from the NPR and supplemented with medication data from the PDR (see online supplemental table 1). Vital status and date of death were provided by the Cause of Death Register. Recent healthcare utilisation for any reason, including hospitalisations and outpatient visits within 1 year before start of follow-up, were identified from the NPR and categorised as 0, 1–3 and ≥4 times separately for inpatient and outpatient visits. Recent use of medications typically used to manage SLE, such as corticosteroids, was assessed from the PDR. The procedure code DT016 (intravenous drug delivery) in the NPR was a crude proxy for these types of treatments, which may include cyclophosphamide, high dose intravenous corticosteroid treatment, intravenous immunoglobulin, rituximab or belimumab. We identified dispensations in the past 6 months when considering medication as a confounder.

### Statistical analysis

Characteristics of the study population were compared by exposure (incident SLE vs general population) using frequencies and means with SD. Crude incidence rates (IR; cases per 1000 person-years) of infection hospitalisation in SLE and the general population were estimated and plotted by time since index date to visualise how the IR changes over time since incident SLE. We estimated crude and adjusted HRs and corresponding 95% CIs for first infection hospitalisation after start of follow-up adjusting for matching factors, education and calendar period (model 1). We further adjusted for comorbidities that might alter infection risk (eg, history of congestive heart disease, atrial fibrillation, hypertension, diabetes mellitus and nephritis) and recent hospitalisations and outpatient visits (model 2). In our third set of adjustments, we added recent corticosteroids, antimalarials, DMARDs or drug infusions (model 3).

For recurrent infection-related hospitalisations, we calculated the number of infection hospitalisations in SLE and the general population during follow-up and the median time (IQR) from index date to event for each. We similarly summarised mortality by SLE status. Within-individual HRs and 95% CIs for recurrent infection hospitalisations were estimated by adjusted frailty models. Similar to above, models were successively adjusted for matching factors, comorbidities and treatment history.

Among individuals with SLE, we calculated IRs by exposure. Using medication as a time-dependent exposure, we estimated the HR and 95% CI for first serious infection associated with starting a DMARD compared with HCQ, adjusted for potential confounders determined *a priori* (age, sex, recent glucocorticoid use, recent drug infusion, history of nephritis, recent infection). Individuals could contribute non-overlapping person-time to both the HCQ-exposed and DMARD-exposed groups. Using similar models, among first DMARD users, we compared azathioprine and MMF separately versus methotrexate. Individuals could only contribute person-time to one group in the head-to-head DMARD analysis, as these were restricted to first ever DMARD initiators. In this analysis, individuals contributed person-time even after they discontinued this DMARD. When examining DMARD exposure in relation to HCQ or in DMARD versus DMARD comparisons, previous or concomitant exposure to HCQ was not considered. If a person was exposed to both DMARD and HCQ, the infectious event was assigned only to the DMARD group. In sensitivity analyses, person-time was censored on the second DMARD dispensation date (switchers) or drug infusion date during follow-up using inverse probability censoring weighting. Because we restricted to individuals who met our register-based criteria for SLE (≥2 visits with ≥1 with a specialist) and then identified first treatment after inclusion date, many patients were excluded who started a medication before reaching these criteria. To examine whether this affected our results, we included treatment initiations up to 90 days before reaching inclusion criteria in a sensitivity analysis with follow-up starting on first treatment dispensation.

## Results

### Incident SLE and general population comparators

Between 2006 and 2013, we identified 2378 individuals with incident SLE who were matched to 11 774 general population comparators. The median time between first SLE-coded visit to inclusion into the cohort was 2.4 months (IQR=0.8, 6.4). The average age was 49 years at start of follow-up, 85% were female and the majority were born in Sweden. As expected, the individuals with SLE had more hospitalisations and outpatient visits in the year prior to start of follow-up and more comorbidities, with the exception of diabetes mellitus which was 5% in both groups. Only 1% of the general population was hospitalised for an infection in the year before start of follow-up compared with 7% of those with SLE (table 1).

**Table 1:** Characteristics of the study population at start of follow-up

	SLE (n=2378)	General population (n=11 774)
Age, mean (SD)	49 (17.6)	49 (17.5)
Female, %	84.5	84.4
Region of residence, %		
Stockholm	21.0	20.4
Uppsala-Örebro	21.6	21.6
West	18.1	18.2
South	17.5	17.5
Southeast	14.1	14.3
North	7.7	8.0
Born in Sweden, %	78.7	85.5
Education, %		
≤9 years	25.9	21.0
10–12 years	41.8	42.3
≥13 years	30.5	32.8
Missing	1.9	3.9
Calendar period, %		
2006‒2009	44.9	45.0
2010‒2013	55.1	55.0
Hospitalisations in the past year, %		
0	58.1	90.5
1–3	36.5	9.1
≥4	5.4	0.4
Outpatient visits in the past year, %		
0	6.2	61.3
1–3	44.3	29.9
≥4	49.5	8.8
History of comorbidity, %		
Congestive heart disease	4.0	1.7
Atrial fibrillation	4.6	2.4
Hypertension	40.5	21.7
Diabetes mellitus	5.3	4.8
Nephritis	15.3	0.7
Serious infection in the past year	7.4	0.9
Medication use*, %		
Systemic corticosteroids	52.1	3.0
Antimalarials	37.9	0.2
DMARDs	18.8	1.0
Biologics	2.0	0.2
Intravenous drug infusion procedure code	4.2	0.5

Percentages may not add up to 100 owing to rounding.

Start of follow-up was the date the individuals with SLE reached inclusion criteria (≥2 visits listing the International Classification of Diseases code for SLE with ≥1 visit with a specialist) and the corresponding date for their matched comparators.

*In the past 6 months, except biologics that is ever used. DMARDs include methotrexate, azathioprine, leflunomide, ciclosporin, cyclophosphamide, mycophenolic acid derivatives, sulfasalazine and tacrolimus.

DMARD, disease-modifying antirheumatic drug.

**Figure 1:**
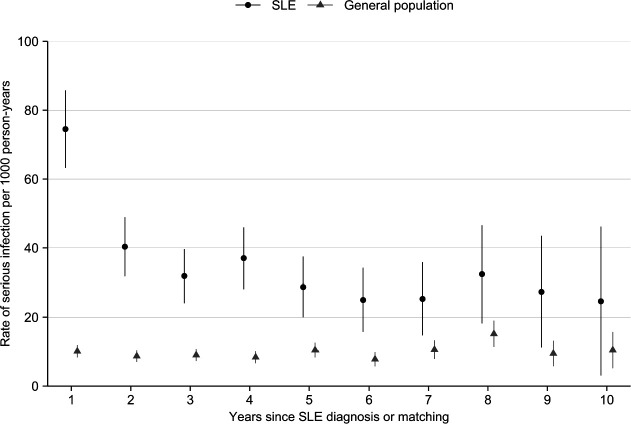
Rates of first serious infection per 1000 person-years in individuals diagnosed with SLE and general population comparators without SLE matched on birth year, sex and residential location, by years since SLE diagnosis or matching.

Over a median 6.2 years of follow-up in the SLE group (95% CI 6.1 to 6.4) and 6.5 years of follow-up in the general population (95% CI 6.4 to 6.5), infection was more common in the individuals with SLE (22% vs 6%). Pneumonia was approximately one-quarter of these infections in both groups (table 2). In SLE, we observed approximately 40 serious infections per 1000 person-years compared with 10 per 1000 person-years in the general population (rate difference, 95% CI 30.1 serious infections per 1000 person-years (26.7 to 33.8); table 2). In multivariable-adjusted models, the rate of first serious infection was significantly higher in the individuals with SLE compared with the general population in all model iterations, which persisted, though attenuated, after adjusting for recent medication use (HR=1.88, 95% CI 1.58 to 2.23; table 2). In SLE, the IR of serious infection was highest in the first year of follow-up and remained consistently higher than the IR in the general population throughout follow-up (figure 1).

**Table 2:** Rates, rate differences and rate ratios for first serious infection after start of follow-up in the SLE and the general population groups (2006–2016)

	SLE (n=2378)	General population (n=11 774)
Serious infection, n (%)		
Overall	511 (21.5)	716 (6.1)
Pneumonia	133 (5.6)	181 (1.5)
Opportunistic	17 (0.7)	9 (0.1)
Sepsis	44 (1.9)	29 (0.2)
Analyses for overall serious infection		
Person-years of follow-up	12 884	74 293
Rate of serious infection per 1000 person-years (95% CI)	39.8 (36.4 to 43.3)	9.6 (7.9 to 11.8)
Rate difference per 1000 person-years (95% CI)	30.1 (26.7 to 33.8)	1.0 (reference)
HR* (95% CI)		
Crude	4.08 (3.65 to 4.58)	1.0 (reference)
Model 1	4.11 (3.66 to 4.61)	1.0 (reference)
Model 2	2.13 (1.85 to 2.46)	1.0 (reference)
Model 3	1.88 (1.58 to 2.23)	1.0 (reference)

*Estimated by Cox proportional hazards models with years since start of follow-up as the underlying time scale (date filled criteria for SLE or the corresponding date for comparators). Model 1 was adjusted for age, sex, region of residence, birth country, education and calendar period. Model 2 was further adjusted for history of congestive heart disease, atrial fibrillation, hypertension, diabetes mellitus, nephritis and the number of hospitalisations and outpatient visits within 1 year before start of follow-up. Model 3 was additionally adjusted for use of systemic corticosteroids, antimalarials, DMARDs and infusions in the hospital within 6 months before start of follow-up.

DMARD, disease-modifying antirheumatic drug.

### Recurrent infections

Eight per cent of patients with SLE had multiple infections during follow-up compared with 1% in the general population. There were 937 total infection hospitalisations among the 2378 individuals with SLE and 936 such infections among the 11 774 individuals from the general population. The rate of recurrence was consistently higher in patients with SLE and these occurred closer to each other (online supplemental table 2). In fully adjusted shared frailty models, patients with SLE had a twofold increased risk of recurrent serious infection (HR=2.22, 95% CI 1.93 to 2.56).

### Infections after medication exposure in SLE

Among the individuals with SLE during follow-up, there were 392 initiations of HCQ vs 387 initiations of azathioprine, MMF or methotrexate between 2006 and 2016. Time since inclusion was longer in DMARD initiators (1.0 years, SD=1.7) compared with HCQ initiators (0.7 years, SD=1.4). History of nephritis and infection was higher among the DMARD initiators, with a higher proportion dispensed corticosteroids (75% vs 44%) or receiving a drug infusion procedure (23% vs 7%) in the 6 months before medication initiation. HCQ initiation was associated with a lower rate of serious infection (32 infections per 1000 person-years) compared with DMARD initiation (57 per 1000 person-years of DMARD exposure). Overall, DMARD initiation was associated with a higher rate serious infection compared with HCQ (HR=1.82, 95% CI 1.27 to 2.60), which decreased after adjustment for history of nephritis, infection and corticosteroid use (HR=1.30, 95% CI 0.86 to 1.95) (table 3).

**Table 3:** Characteristics of individuals with SLE initiating hydroxychloroquine and disease-modifying drugs after start of follow-up*, incidence rates of serious infections and HR with 95% CI comparing DMARD initiators with hydroxychloroquine initiators

	Hydroxychloroquine initiators	DMARD initiators
N	392	387
Age, mean (SD)	46.7 (17.4)	45.9 (18.1)
Female, %	87.5%	80.1%
Years since SLE index date*, median (IQR)	0.10 (0.01, 0.75)	0.41 (0.09, 1.06)
History of nephritis	15.6%	39.5%
History of serious infection <1 year before start	7.7%	12.4%
Corticosteroid dispensation <6 months before start	44.4%	74.7%
Intravenous drug procedure code <6 months before start	6.9%	23.3%
Serious infections/person-years	45 cases/1396 person-years	94 cases/1665 person-years
Incidence rate (95% CI) per 1000 person-years	32.2 (24.1 to 43.2)	56.5 (46.1 to 69.1)
HR (95% CI) age-adjusted and sex-adjusted	1.0 (reference)	1.82 (1.27 to 2.60)
HR (95% CI) adjusted for nephritis, corticosteroids, history of infusion, history of infection	1.0 (reference)	1.30 (0.86 to 1.95)

*Start of follow-up was when inclusion criteria were met (≥2 ICD coded visits with ≥1 ICD code from a specialist).

DMARD, disease-modifying antirheumatic drug; ICD, International Classification of Diseases.

There was considerable heterogeneity across the three major DMARDs. MMF initiators were more likely to have a history of nephritis (71%), recent corticosteroid use (78%), recent drug infusion (43%) and a serious infection in the past year (16%). The IR of serious infection was lowest following methotrexate initiation (32 infections per 1000 person-years) and highest for azathioprine initiators (71 per 1000 person-years). Azathioprine use was associated with a twofold higher rate of infection in adjusted models compared with methotrexate (fully adjusted HR 2.19, 95% CI 1.14 to 4.21). MMF initiators had an infection rate of 50 per 1000 person-years and adjusted HR of 1.39 (95% CI 0.65 to 2.96; table 4).

**Table 4:** Characteristics of individuals with SLE initiating methotrexate, azathioprine and mycophenolate mofetil as their first DMARD after SLE diagnosis, incidence rates of serious infections and HR with 95% CI comparing azathioprine and mycophenolate mofetil initiators with methotrexate initiators

	Methotrexate	Azathioprine	Mycophenolate
N	76	191	120
Age, mean (SD)	49 (18)	45.9 (18.6)	42 (17)
Female, %	88.2%	80.1%	75.0%
Years since SLE index date, median (IQR)	0.92 (0.24, 2.15)	0.43 (0.08, 1.01)	0.27 (0.07, 0.57)
History of nephritis	11.8%	30.9%	70.8%
History of serious infection <1 year before start	9.2%	11.5%	15.8%
Corticosteroid dispensation <6 months before start	64.5%	76.4%	78.3%
Intravenous drug procedure code <6 months before start	5.3%	18.3%	42.5%
Serious infections/person-years	11/346	58/817	25/502
Incidence rate (95% CI) per 1000 person-years	31.8 (17.6 to 57.5)	71.0 (54.9 to 91.8)	49.8 (33.7 to 73.7)
HR (95% CI) age-adjusted and sex-adjusted	1.0 (reference)	2.31 (1.21 to 4.40)	1.79 (0.88 to 3.66)
HR (95% CI) adjusted for nephritis, corticosteroids, history of infusion, history of infection	1.0 (reference)	2.19 (1.14 to 4.21)	1.39 (0.65 to 2.96)

DMARD, disease-modifying antirheumatic drug.

### Sensitivity analyses

When allowing for treatment initiations within the 3 months prior to reaching the SLE inclusion criteria, the proportion of patients with a history of comorbidity, corticosteroid dispensations and drug infusions decreased for all treatment groups. The fully adjusted HR comparing DMARD with HCQ was slightly higher than in the primary analyses (HR=1.69, 95% CI 1.26 to 2.27; online supplemental table 3). The HR for azathioprine compared with methotrexate was lower (HR=1.70 95% CI 1.11 to 2.60) and the HR for MMF was slightly higher (HR=1.50 95% CI 0.92 to 2.47).

Additional sensitivity analyses censoring on switching to another DMARD (n=81) or drug infusion procedure during follow-up (n=67), yielded comparable results. Additionally, accounting for potential selection bias using inverse probability censoring weights did not appreciably alter the interpretation but did increase the estimates for both azathioprine and mycophenolate versus methotrexate (online supplemental table 4).

## Discussion

Individuals recently diagnosed with SLE were two to four times more likely to be hospitalised with an infection and to have recurrent serious infections compared with age-matched and sex-matched general population comparators. The rate of infection was pronounced in the year following diagnosis and remained elevated for the study period compared with the general population, which raises the question of whether less controlled disease and disease activity may influence underlying infection risk. The incidence rate of infection was higher following DMARD initiation compared with HCQ initiation among individuals with incident SLE. We found some heterogeneity by DMARD with azathioprine initiators consistently experiencing more infection hospitalisations compared with methotrexate.

The immune-modulating and immunosuppressive aspects of SLE and its treatment have been implicated in higher infection risk in SLE cohorts.^9–11^ A recent population-based study of incident SLE found comparable higher rates of first severe infection (relative risk =1.82, 95% CI 1.66 to 1.99) compared with matched general population comparators.^8^ A recent meta-analysis examined infection risk by comorbidity and treatment, combining dozens of heterogeneous studies. The majority of work in this area has focused on prevalent SLE, prevalent medication use, treatment history or some combination of these.^4 11 16^ In contrast, our population-based work focuses on incident SLE and incident medication use.

Initiators of azathioprine and MMF had higher rates of serious infection than methotrexate. Underlying features of a patient’s SLE likely contribute to their treatment options. For example, nephritis is a severe SLE manifestation and a primary indicator for MMF in Sweden. We found that nearly 71% of those initiating this drug had a history of nephritis, compared with 12% and 31% in methotrexate and azathioprine, respectively. Such channelling of patients may lead to confounding by indication. We accounted for differences in severity, disease activity, SLE phenotype and comorbidity to the best of our ability via multivariable-adjustment including, but not limited to, covariates such as corticosteroid use and recent healthcare utilisation. Accounting for potential confounding by corticosteroid use is challenging given how this medication is dispensed and used to manage SLE; therefore, there may be some residual confounding by steroid dose. Further measures of disease activity are not available in these types of register data. Additional adjustment attenuated the mycophenolate-infection association, however the increased infection rate associated with azathioprine initiation persisted. Given there may be regional differences in how DMARDs are prioritised, the generalisability of our findings apply to regions with similar channelling of these medications.

We did not have data on methylprednisolone pulse treatment specifically or intravenous antibiotics in this register linkage. We were limited in our ability to identify specific infusion therapies when censoring and applied a crude definition based on infusion procedures, which may lead to some misclassification. Given that our study population is incident SLE and infusions are more likely to be a third-line treatment, we anticipate this is not a big limitation. When also censoring at infusion procedure after initiation of DMARD, the findings were consistent.

Power was limited to sufficiently explore the role of infusions, particularly with respect to the MMF initiators. The ALMS trial demonstrated that mycophenolate can be used with equal efficacy as induction treatment for nephritis compared with cyclophosphamide, which altered how patients with nephritis were managed starting around 2011 (eg, less intravenous cyclophosphamide).^17^ We tried to determine whether preceding infusion might similarly increase infection risk differentially in the mycophenolate group but found that even when stratifying by history of infusion within 6 months before mycophenolate start, the incidence rates were similar (50.3 infections/1000 person-years without infusion vs 49.1 infections/1000 person-years with infusion). We cannot rule out whether perceived infection risk modified patient behaviour which drove differences in infection risks.

We applied a new-user design to this register-based cohort study generally representing the entire Swedish population; however, there may be some misclassification. We used a minimum of 5 years of outpatient and inpatient data to identify and exclude prevalent SLE. Without primary care data, some prevalent SLE may be misclassified as incident. However, even patients with well-controlled SLE are recommended to see their rheumatologist annually, therefore this misclassification is likely minor. Some proportion of patients were on treatment before satisfying the SLE definition, which is due to our SLE case definition and reflects the diagnostic process. In sensitivity analysis extending the new-user time window, results were similar. Few DMARD initiators (n=37) had HCQ dispensed on the same day, and 207 DMARD initiators had a history of HCQ dispensing in the prior 6 months. The use of person-time and time-dependent exposure accounted for this in analyses.

Infection risk in patients with autoimmune disease and on immunosuppressants is of great interest. Briefly, among first infection hospitalisations following SLE diagnosis, we found 133 infections attributed to pneumonia (5.6% of the incident SLE population) in contrast to 1.5% of the general population. Given the availability of immunisations for pneumonia and a considerable proportion of pneumonia infections in these patients with lupus, future work needs to build on past work showing low vaccination rates^18^ to further examine vaccine uptake and effectiveness in this population, and examine antibody response following vaccination. Interestingly, initiation of azathioprine was consistently associated with increased rates of serious infection in this register-based inception cohort compared with methotrexate initiators, which may warrant additional counselling about infection-related risks in these patients.

## Data Availability

Data may be obtained from a third party and are not publicly available. Data may be obtained from a third party (Socialstyrelsen and Statistics Sweden) and are not publicly available.
