# Supplementary material for: Infection hospitalisation in systemic lupus in Sweden
Source: Lupus Sci Med. 2021 Sep 14;8(1):e000510. doi: 10.1136/lupus-2021-000510 (PMC8444249; doi:10.1136/lupus-2021-000510)
Supplement: Supplementary data [file lupus-2021-000510supp001.pdf]

Supplemental Table 1. Definitions of exposure, outcome, and covariates used in analyses including International Classification of Diseases (ICD) Swedish revision codes and Anatomical Therapeutic Chemical (ATC) classification system codes.

**Variable**	**Definition**	**ICD/ATC codes***
Systemic lupus erythematosus (exposure)	≥2 inpatient or outpatient visits in the NPR with ≥1 visit in a rheumatology, internal medicine, nephrology, dermatology, or pediatric clinic	*ICD-10:* M32.1–M32.9<br>*ICD-9:* 710A<br>*ICD-8:* 734,1
Serious infection (outcome)	An inpatient visit in the NPR for which infectious disease was the primary discharge diagnosis	*ICD-10:* A00–B99; D73.3; E06.0; E32.1; G00–G02; G05–G07; G04.2; H00.0; H44.0; H60.0–H60.3; I30.1; I40.0; J00–J22; J32; J34.0; J36; J38.3; J39.0; J39.1; J44.0; J85; J86; K04.4; K04.6; K04.7; K10.2; K11.3; K12.2; K14.0; K57.0; K57.2; K57.4; K57.8; K61; K63.0; K65.0; K65.9; L00–L08; L30.3; M00; M01; M46.2; M46.3; M46.4; M64.5; M60.0; M65.0; M71.0; M71.1; M72.6; M86; N10–N12; N13.6; N15.1; N15.9; N30.0; N30.8; N34; N39.0; N41.2; N43.1; N45; N48.2; N61; N70–N74; N75.1; O23; O26.4; O41.1; O75.3; O85; O86; O88.3; O91; O98; H66; H67; H70
Pneumonia	An inpatient visit in the NPR for which pneumonia was the primary discharge diagnosis	*ICD-10:* J11.0; J12–J18
Opportunistic infections	An inpatient visit in the NPR for which an opportunistic infection was the primary discharge diagnosis	*ICD-10:* A02; A15–A19; A31; A32; A43; A48.1; A81.2; B02; B38; B39; B40; B44; B45; B58; B59
Sepsis	An inpatient visit in the NPR for which sepsis was the primary discharge diagnosis	*ICD-10:* A40; A41
Congestive heart disease	≥1 visit in the NPR before inclusion	*ICD-10:* I42; I50<br>*ICD-9:* 425; 428<br>*ICD-8:* 425; 427,0; 427,1; 428,9
Atrial fibrillation	≥1 visit in the NPR before inclusion	*ICD-10:* I48<br>*ICD-9:* 427D<br>*ICD-8:* 427,92
Hypertension	≥1 visit in the NPR or ≥1 dispensation in the PDR before inclusion	*ICD-10:* I10–I15<br>*ICD-9:* 400–405<br>*ICD-8:* 400–404<br>*ATC:* C07–C09
Diabetes mellitus	≥1 visit in the NPR or ≥1 dispensation in the PDR before inclusion	*ICD-10:* E10; E11<br>*ICD-9:* 250<br>*ICD-8:* 250<br>*ATC:* A10
Nephritis	≥1 visit in the NPR before inclusion	*ICD-10:* N00–N06; N16.2; N16.4; N16.8; N17–N19; N26; M32.1
Systemic corticosteroids	≥1 dispensation in the PDR within 3 months before inclusion	*ATC:* H02AB01; H02AB04; H02AB06; H02AB07
Antimalarials	≥1 dispensation in the PDR within 3 months before inclusion	*ATC:* P01BA01; P01BA02

Disease modifying antirheumatic drugs (DMARDs)	≥1 dispensation in the PDR within 3 months before inclusion	*ATC:* A07EC01; L01AA01; L01BA01; L04AA06; L04AA13; L04AD01; L04AD02; L04AX01; L04AX03
Biologics	≥1 dispensation in the PDR or ≥1 visit in SRQ denoting medication use before inclusion	*ATC:* L01XC02; L01XC10; L04AA24; L04AA26; L04AB01; L04AB02; L04AB04; L04AB05; L04AB06; L04AC03; L04AC05; L04AC07; L04AC10
Drug infusion	≥1 visit in the NPR before inclusion	*KVÅ:* DT016

ICD = International Classification of Diseases; ATC = Anatomical Therapeutic Chemical; KVÅ = Klassifikation av vårdåtgärder (classification of healthcare interventions); NPR = National Patient Register; PDR = Prescribed Drug Register; SRQ = Swedish Rheumatology Quality Register.
*Codes include all subcategories in the classification system.

Supplemental Table 2. Analysis of recurrent serious infections and death during follow-up in SLE and the general population comparators. Hazard ratios and 95% confidence intervals from shared frailty models.

	**SLE** (n=2378)		**General population** (n=11 774)	
	**n (%)**	**Years from inclusion to event, median (IQR)**	**n (%)**	**Years from inclusion to event, median (IQR)**
Serious infection*				
0	1869 (78.6)	—	11 059 (93.9)	—
1	310 (13.0)	2.1 (0.7, 4.0)	585 (5.0)	3.4 (1.6, 5.7)
2	103 (4.3)	2.7 (1.5, 5.0)	80 (0.7)	4.4 (2.5, 6.7)
3	44 (1.9)	3.6 (2.5, 5.7)	31 (0.3)	4.9 (3.5, 6.6)
4	22 (0.9)	4.2 (2.9, 5.5)	5 (<0.1)	6.2 (4.0, 7.9)
5	11 (0.5)	4.7 (3.2, 5.8)	8 (0.1)	7.4 (5.6, 8.8)
Death	280 (8.4)	3.2 (1.2, 5.4)	544 (4.3)	4.2 (2.4, 6.1)

**Shared Frailty Models**		
Crude Model, HR (95% CI)	5.24 (4.78, 5.75)	1.0 (reference)
Model 1, HR (95% CI)	5.24 (4.77, 5.76)	1.0 (reference)
Model 2, HR (95% CI)	2.64 (2.35, 2.98)	1.0 (reference)
Model 3, HR (95% CI)	2.22 (1.93, 2.56)	1.0 (reference)

HR = hazard ratio; CI = confidence interval; IQR = interquartile range.
*A few individuals had more than five recurrent infections (SLE, n=19, general population, n=6). For secrecy reasons, categories with less than five individuals were not presented.
**Estimated by Cox proportional hazards models with years since inclusion as the time scale. Model 1 was adjusted for age, sex, region of residence, birth country, education, and calendar period. Model 2 was further adjusted for history of congestive heart disease, atrial fibrillation, hypertension, diabetes mellitus, nephritis, and number of hospitalizations and outpatient visits one year before start of follow-up. Model 3 was additionally adjusted for use of systemic corticosteroids, antimalarials, DMARDs, and infusions in the hospital within six months before start of follow-up.

Supplemental Table 3. Characteristics of individuals with SLE initiating hydroxychloroquine and disease modifying drugs *allowing treatment to occur within 90 days before 2nd SLE ICD-coded visit (index date)**, incidence rates of serious infections and hazard ratio with 95% confidence interval comparing DMARD initiators to hydroxychloroquine initiators.

	**HCQ**	**DMARD**	**Methotrexate**	**Azathioprine**	**Mycophenolate**
N	661	819	205	404	210
Age, mean (SD)	45.3 (17.2)	44.6 (17.1)	45.2 (16.4)	44.1 (17.3)	41.1 (16.7)
Female, %	88.2%	84.0%	87.8%	84.9%	78.6%
Years since SLE index date*, median (IQR)	0 (-0.08, 0.20)	0.30 (0.01, 1.17)	0.50 (0.02, 2.89)	0.28 (0.01, 1.03)	0.18 (0.00, 0.55)
History of nephritis	12.7%	30.9%	9.8%	24.5%	63.8%
History of serious infection <1 yr before start	6.4%	8.6%	5.4%	8.4%	11.9%
Corticosteroid dispensation <6 mo before start	39.2%	72.0%	67.3%	73.8%	73.3%
IV drug infusion procedure code <6 mo before start	4.8%	16.0%	3.9%	13.4%	32.9%
Serious infections/person-years	68/2368	173 /3523	28 /888	98 /1729	47 /906
Incidence rate (95% CI) per 1000 pyr	28.7 (22.6, 36.4)	49.1 (42.3, 57.0)	31.5 (21.8, 45.7)	56.7 (46.5, 69.1)	51.9 (39.0, 69.1)
HCQ vs. DMARD Model 1** HR (95% CI)	1.0 (reference)	1.92 (1.45, 2.55)			
HCQ vs. DMARD Model 2^ HR (95% CI)	1.0 (reference)	1.69 (1.26, 2.27)			
DMARD vs. MTX Model 1** HR (95% CI)			1.0 (reference)	1.76 (1.15, 2.68)	1.80 (1.12, 2.89)

DMARD vs. MTX<br>Model 2^ HR (95% CI)			1.0 (reference)	1.70 (1.11, 2.60)	1.50 (0.92, 2.47)

*SLE index date was the date inclusion criteria were reached (2nd ICD coded visit with at least one from a specialist).

** Model 1 Age & sex-adjusted

^ Model 2 additionally adjusted for nephritis, corticosteroids, history of infusion, history of infection

Supplemental Table 4. Incidence rates of serious infections and hazard ratio with 95% confidence interval comparing azathioprine and mycophenolate mofetil initiators to methotrexate initiators, *censoring on start of another DMARD or infusion during follow-up*. Hazard ratios were estimated from 1) a traditional Cox model including DMARD switch date and infusion as censoring dates and 2) a weighted Cox model in which inverse probability of censoring weights were used to account for censoring on DMARD switching.

	**Methotrexate**	**Azathioprine**	**Mycophenolate**
N	76	191	120
Serious infections / person-years	7 cases/254 person-years	44 cases/578 person-years	15 cases/348 person-years
Incidence rate (95% CI) per 1000 pyr	27.6 (95%CI 13.1, 57.8)	78.1 (95%CI 56.6, 102.2)	43.1 (95%CI 26.0, 71.6)
1) HR (95% CI) Adj for nephritis, corticosteroids, history of infusion, history of infection	1.0 (reference)	2.82 (1.27, 6.28)	1.73 (0.70, 4.26)
2) IPCW weighted Cox model HR (95% CI) Adj for nephritis, corticosteroids, baseline history of infection and baseline history of infusion	1.0 (reference)	3.15 (1.40, 7.08)	2.22 (0.77, 6.39)

Supplementary Table 5. Incidence rates of serious infections and hazard ratio with 95% confidence interval comparing azathioprine and mycophenolate mofetil initiators to methotrexate initiators stratified by year (<2011 vs. ≥2011).

	Methotrexate		Azathioprine		Mycophenolate	
	**<2011**	**≥2011**	**<2011**	**≥2011**	**<2011**	**≥2011**
N	35	41	98	93	54	66
Serious infections / person-years	6 cases/229 pyrs	5 cases/117 pyrs	37 cases/536 pyrs	21/282 pyrs	14 cases/286 pyrs	11 cases/216 pyrs
Incidence rate per 1000 pyr (95% CI)	26.2 (95%CI 11.8, 58.4)	42.8 (95%CI 17.8, 102.9)	69.1 (95%CI 50.0, 95.3)	74.6 (95%CI 48.6, 114.4)	48.9 (95%CI 29.0, 82.6)	51.0 (95%CI 28.2, 92.0)
HR (95% CI) Age & sex-adjusted	1.0 (reference)	1.0 (reference)	2.5 (1.0, 5.9)	2.4 (0.9, 6.5)	2.0 (0.8, 5.2)	1.7 (0.6, 4.9)
HR (95% CI) Adj for nephritis, corticosteroids, history of infusion, history of infection	1.0 (reference)	1.0 (reference)	2.2 (0.9, 5.4)	2.1 (0.8, 5.7)	1.39 (0.66, 2.97)	0.9 (0.3, 3.0)
